# Normative Scores for CrossFit^®^ Open Workouts: 2011–2022

**DOI:** 10.3390/sports11020024

**Published:** 2023-01-18

**Authors:** Gerald T. Mangine, Nina Grundlingh, Yuri Feito

**Affiliations:** 1 Department of Exercise Science and Sport Management, Kennesaw State University, Kennesaw, GA 30144, USA; 2 Department of Data Science and Analytics, Kennesaw State University, Kennesaw, GA 30144, USA; 3 American College of Sports Medicine, Indianapolis, IN 46202, USA

**Keywords:** fitness assessment, sport specific, athlete classification, high-intensity functional training, sex differences

## Abstract

To create normative scores for all CrossFit^®^ Open (CFO) workouts and compare male and female performances, official scores were collected from the official competition leaderboard for all competitors of the 2011–2022 CFO competitions. Percentiles were calculated for athletes (18–54 years) who completed all workouts within a single year ‘as prescribed’ and met minimum scoring thresholds. Independent t-tests revealed significant (*p* < 0.05) sex differences for 56 of 60 workouts. In workouts scored by repetitions completed, men completed more repetitions in 18 workouts by *small* to *large* differences (*d* = 0.22–0.81), whereas women completed more repetitions in 6 workouts by *small* to *medium* differences (*d* = 0.36–0.77). When workouts were scored by time to completion, men were faster in 10 workouts by *small* to *large* differences (*d* = 0.23–1.12), while women were faster in 3 workouts by *small* differences (*d* = 0.46). In three workouts scored by load lifted, men lifted more weight by *large* differences (*d* = 2.00–2.98). All other differences were either *trivial* or not significant. Despite adjusted programming for men and women, the persistence of performance differences across all CFO workouts suggests that resultant challenges are not the same. These normative values may be useful for training and research in male and female CrossFit^®^ athletes.

## 1. Introduction

The CrossFit^®^ Open (CFO) has been the initial qualifying round for the CrossFit Games^TM^ competition since 2011 [1]. It has typically consisted of 3–6 workouts that variably challenge some aspect related to an athlete’s strength, endurance, sport-specific skill, or a combination of these [2,3]. Heading into the competition each year, athletes are aware of the number of weeks the CFO will last (3–5 weeks) but are unaware of each workout’s specific details until they are individually released via online broadcast each Thursday evening. Since competitors are only given four days to complete a given week’s workout and submit their best score to competition officials [3], they should ideally be prepared for all possibilities.

It is known that each CFO battery will consist of a unique set of workouts, all formatted to produce a score that readily distinguishes performance [2,3]. Athletes have been challenged with completing a list of exercises as quickly as possible and were ranked by time to completion (TTC), and at times, the TTC of certain tie-breaking criteria. Approximately 90% of TTC-style workouts have also been assigned a time limit [2,3], and for these, athletes who did not finish all the workout when the time expired were scored by the number of repetitions they completed. The most common format, however, assigned a list of exercises to be completed for ‘as many repetitions as possible’ (AMRAP) within a time limit, and athletes were ranked by the total number of repetitions they completed. Out of the 60 scored CFO workouts programmed between 2011 and 2022, 35 have been AMRAP-style events. Very rarely (~5% of CFO workouts), athletes were tasked with finding their one-repetition maximum (1-RM) in a single exercise or complex within a time limit, and performance was based on load lifted.

Although CFO workouts might be limited in structure, and workouts have consistently included certain exercises from year to year, there are infinite possible exercise–prescription combinations. Each combination may uniquely challenge one or more energy systems and require different degrees of technical skill as well as strength and power. The CFO is indeed an accurate representation of the CrossFit^®^ ideology which aims for simultaneous improvements in all areas of fitness [4]. In support of this, most investigated measures of body composition [5,6,7], strength and power [6,8,9,10], and aerobic and anaerobic capacity [5,6,8,9,10,11,12] have been related to performance. Athletes might use normative scores for many of these traditional physiological measures to estimate their ability to perform in competition [13]. However, the reported relationships have not demonstrated a hierarchal order of importance, and this is likely because they were not founded upon consistency. Sample populations, methods used for collecting physiological measures, and the workouts used to define performance have all varied across studies, leaving little clarity as to which laboratory-based measures should be monitored during training to predict competition performance. Further, it may not be practical or logistically feasible for non-researchers to acquire the expensive equipment (e.g., metabolic cart, cycle ergometers, force plates) needed to perform many traditional assessments. Standardized methods require varying degrees of expertise, are not always conducive for testing large groups, and are likely to impair movement and transitions if the desire is to measure responses during a typical CrossFit^®^ workout.

Another solution may be to utilize CrossFit^®^-style workouts themselves to track progress and predict competition performance. Logically, performing well in these workouts during training or competition should be a strong predictor of future CFO performances. Indeed, past rankings at various stages of the CrossFit Games^TM^ competitions have been shown to be indicative of 2020 CFO performance [14], and self-reported scores in benchmark workouts have also been shown to variably distinguish performance in 2016 [15] and 2018 [6] CFO competitors. Typically, any exercise and prescription combination could be programmed on any given training day [4], and this lack of consistency is problematic for tracking progress. However, benchmark workouts are unique because they are readily identified by their name (e.g., Fran, Grace, Murph) and their prescription is standard. After their initial introduction, CFO workouts become benchmark workouts and may periodically be programmed into normal training and have even reappeared in later CFO competitions [2,16]. By monitoring their performance in these workouts, and relating it to a specific percentile rank, athletes might gauge how they would perform in future CFO competitions.

Thus far, normative scores have only been published for five benchmark workouts (i.e., Grace, Fran, Helen, Fight-Gone-Bad, and Filthy-50) [17]. These were chosen because CFO athletes are able to self-report their performances for these specific workouts to their user profile on the official CrossFit Games^TM^ leaderboard [18]. However, because scores are self-reported, performances are not verified, and scores may be updated at any time, their veracity and timeliness are questionable. In contrast, CFO workout performances must meet specific criteria to appear on the leaderboard [3,18]. For instance, athletes must either complete the workout at a CrossFit^®^-affiliated gym or in front of a judge who has passed the judges’ certification course and who certifies that the athlete met all workout requirements and movement standards. Alternatively, athletes may submit a video recording of their performance using specific filming criteria and competition officials perform the judge’s task. Because submissions are only accepted, validated, and ranked if they are received within the 4-day window following each workout’s release, confidence in their accuracy and timeliness is much higher. While each competitor receives an official rank (absolute and percentile) for each validated submission, the separation between scores of neighboring percentile ranks is not made clear. Workout percentile ranks may also vary weekly for reasons other than differences in workout prescription, for example, as specialists, scaled athletes, and injured athletes join or leave the main competition (i.e., report or fail to submit their scores). Therefore, the purpose of this study was to create normative scores for all existing CFO workouts (i.e., from 2011 to 2022) using official scores of competitors who completed each workout as prescribed (i.e., Rx) within each respective competition year. Additionally, because workouts are most often programmed differently between men and women, a secondary aim was to examine sex differences in the performance of each workout.

## 2. Materials and Methods

### 2.1. Experimental Design

Performance data were collected for all athletes participating in CFO competitions from 2011 to 2022. All competition results were obtained from the JSON file located on the publicly available, official competition leaderboard [19]. Python3 was used to convert the data into a CSV format, and the data were treated in Microsoft Excel (v. 365, Microsoft Corporation, Redmond, VA, USA). Since these data were pre-existing and publicly available, the University’s Institutional Review Board classified this study as exempt, which did not require athletes to provide their informed consent (IRB #16-215). Treating the data involved removing all age-group athletes (e.g., teens and masters) and cases that did not meet study inclusion criteria. The retained data included each athlete’s age and final overall ranking (within a given year), as well as their rank and score for every CFO workout that they completed.

### 2.2. Participants

From 2011 to 2022, total CFO participation ranged between 13,127 and 399,538 combined male and female athletes [19]. The entire population for each year included all Rx, scaled, and adaptive athletes from each age grouping, as well as athletes who registered for the competition but did not submit scores for any workouts. For this study, age, rank, and workout performance data (rank and score) were retained for all athletes between the ages of 18 and 54 years (i.e., non-age-group athletes) who completed all CFO workouts as prescribed (i.e., as Rx with no within-sex scaling) within a specific competition year. To limit the inclusion of workout “specialists” and those who did not intend on completing or could not perform the exercises for the Rx workout (e.g., when an athlete completed only a few repetitions of an Rx workout to boost their overall ranking), cases were excluded if any of their scores did not surpass a minimum threshold within a single competition year. The minimum thresholds defined for this study required athletes to complete:

1. At least one round (in AMRAP-style workouts);
2. The first exercise couplet in workouts where couplets were repeated;
3. All repetitions assigned for the first exercise in the list (TTC workouts) or when several rounds were not expected (in AMRAP-style workouts);
4. Timed workouts within 60 min when no time limit was programmed (i.e., CFO 14.5, CFO 15.5, and CFO 16.5);
5. A 1-RM with a load equal to or greater than the standard barbell used by men (45 lbs. (20.4 kg)) and women (35 lbs. (15.9 kg)).

Treating the data set with these criteria produced the total study population for each CFO year. Then, to minimize the effect of reporting or validation errors (intentional or non-intentional), random samples of approximately 68% of athletes from each study population (i.e., equal to approximately ± 1 standard deviation (SD)) [20] were drawn and retained for statistical analyses. Table 1 provides a summary of the initial population of athletes for each year, the number of cases meeting study criteria, and the age and final competition ranking characteristics of each final sample.

### 2.3. Workout Descriptions

Changes to the competition format have occurred throughout the CFO’s history [3]. The competition has always released 1–2 workouts each week on Thursday evenings via live online broadcast, and competitors have always been allotted four days to complete the workout at their normal training facility and upload their best score to the online leaderboard [19]. With a few exceptions, competitors have always been given different instructions for completing Rx (i.e., ‘as prescribed’) and scaled versions of each workout, as well as those prescribed to teen and masters athletes [2,3]. Additional workout versions were programmed in more recent years with the introduction of the adaptive, foundations, and equipment-free divisions. In each instance, the modified workout typically programmed variants of Rx exercises, prescribed different repetition counts (per exercise), and/or different intensity loads when applicable [3]. Because these differences alter the assigned workload, equating different CrossFit^®^-style workouts is inherently difficult [21], and verifying modified workloads may not be possible, only Rx performances were considered for this study. Cases were also excluded if the reported age was not between 18 and 54 years due to the lack of clarity on the leaderboard about which workout version these athletes completed. Otherwise, all retained scores were assumed to have been representative of attempts made using Rx standards.

The data retained for analysis included the athlete’s official rank for each workout and score, recorded as TTC (in minutes), repetitions, or load (in lbs. (kg)). Whenever the score could be officially quantified in multiple units (e.g., CFO 17.1 could be quantified as TTC or repetitions if the workout was not completed within the time limit), all scores were converted into a repetition completion rate (i.e., repetitions completed divided by TTC or workout duration; repetitions · minute^−1^) as previously described [21,22]. In these instances, the calculated repetition completion rate was used for all statistical analyses and to present sex differences, whereas the original scoring format was used to present normative scores.

### 2.4. Statistical Analysis

Statistical software (SPSS, v.28.0, SPSS Inc., Chicago, IL, USA) was used for random sampling, as well as to calculate means, SDs, and percentiles for men and women separately. Independent *t*-tests were used to examine sex differences for each workout. Significance was accepted at an alpha level of *p* ≤ 0.05. Effect sizes (*d*) were also used to quantify the magnitude of any observed differences [23]. Interpretations of effect size were evaluated at the following thresholds: *trivial* (*d* < 0.20), *small* (*d* = 0.20), *medium* (*d* = 0.50), and *large* (*d* ≥ 0.80). All data are reported as mean ± standard error (SE).

## 3. Results

The specific programming details for each workout included in this study are provided alongside their respective normative scores throughout Table 2, Table 3, Table 4, Table 5, Table 6 and Table 7.

### Sex Differences

**Figure 1:**
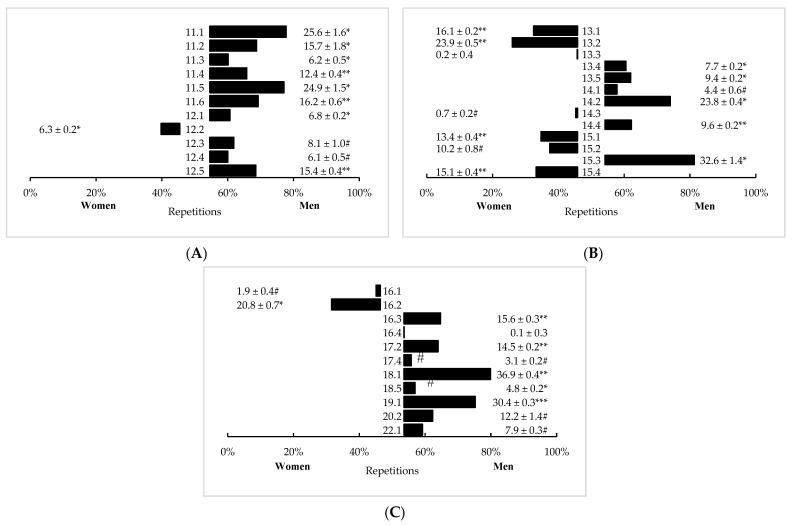
Sex differences in AMRAP-style CFO workouts programmed from (**A**) 2011–2012, (**B**) 2013–2015, and (**C**) 2016–2022 (mean difference ± SE). # = *Trivial*, significant (*p* < 0.05) difference between men and women. * = *Small*, significant (*p* < 0.05) difference between men and women. ** = *Medium*, significant (*p* < 0.05) difference between men and women. *** = *Large*, significant (*p* < 0.05) difference between men and women.

In AMRAP-style workouts, significant (*p* < 0.05) differences between men and women in repetitions completed were observed in 33 (out of 35) workouts. Men outperformed women in 24 of these workouts with 1 by a *large* difference (CFO 19.1, *p* < 0.001, *d* = 0.81), 7 by *medium* differences (*p* < 0.001, *d* = 0.52–0.78), and 10 by *small* differences (*p* < 0.001, *d* = 0.22–0.48). Women completed more repetitions than men in nine workouts with four by *medium* differences (*p* < 0.001, *d* = 0.51–0.77) and two by *small* differences (CFO 16.2, *p* < 0.001, *d* = 0.36; CFO 12.2, *p* < 0.001, *d* = 0.46). Sex differences in all remaining AMRAP-style workouts were either *trivial* or not significant. Mean differences (± SE) between sexes in AMRAP-style workouts are illustrated in Figure 1.

In TTC-style workouts that did not have a time limit, *small* sex differences were noted where women completed CFO 14.5 (mean difference = 1.1 ± 0.1 min, *p* < 0.001, *d* = 0.23) and CFO 16.5 (mean difference = 1.3 ± 0.1 min, *p* < 0.001, *d* = 0.40) faster than men. A significant but trivial difference (*p* < 0.001, *d* = 0.08) was seen between women (10.9 ± 0.1 min) and men (11.1 ± 0.1 min) for CFO 15.5.

**Figure 2:**
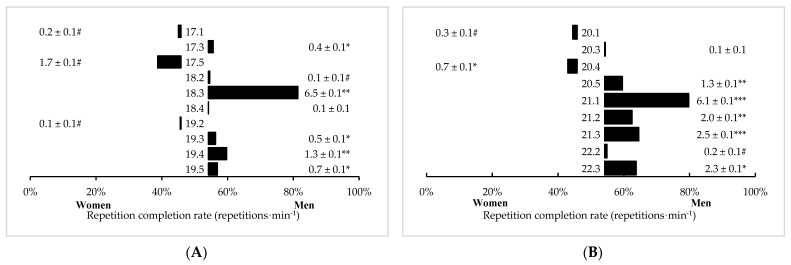
Sex differences in timed TTC-style CFO workouts programmed from (**A**) 2017–2019 and (**B**) 2020–2022 (mean difference ± SE). # = *Trivial*, significant (*p* < 0.05) difference between men and women. * = *Small*, significant (*p* < 0.05) difference between men and women. ** = *Medium*, significant (*p* < 0.05) difference between men and women. *** = *Large*, significant (*p* < 0.05) difference between men and women.

In time-limited TTC-style workouts, significant (*p* < 0.05) differences between men and women in repetition completion rate were observed in 17 (out of 19) workouts. Men completed 12 of these workouts at a faster rate than women, with 2 by *large* differences (CFO 21.1, *p* < 0.001, *d* = 0.92; CFO 21.3, *p* < 0.001, *d* = 1.12), 4 by *medium* differences (*p* < 0.001, *d* = 0.61–0.76), and 4 by *small* differences (*p* < 0.001, *d* = 0.23–0.46). Women completed CFO 20.4 at a faster rate than men, but by a *small* difference (*p* < 0.001, *d* = 0.46). Sex differences in all remaining time-limited TTC-style workouts were either *trivial* or not significant. Mean differences (±SE) between sexes in time-limited TTC-style workouts are illustrated in Figure 2.

In workouts scored by load lifted, *large* sex differences (*p* < 0.001, *d* = 2.00–2.98) where men lifted more weight than women were seen for CFO 15.1a (mean difference = 13.7 ± 0.1 kg), CFO 18.2a (mean difference = 35.4 ± 0.1 kg), and CFO 21.4 (mean difference = 37.3 ± 0.1 kg). Body mass and height were not reported by all participants each year, and due to concerns about the accuracy and timeliness of available information, strength performance differences were not assessed relative to body size.

## 4. Discussion

The purpose of this study was to create normative scores for workouts programmed for the men’s and women’s divisions for the 2011 through 2022 CFO competitions. Objectively tracking progress with CrossFit^®^ training is difficult because workouts vary daily to simultaneously stimulate adaptations in all relevant parameters of fitness [4]. Although any targeted physiological trait can be assessed by a variety of commonly accepted field and laboratory tests [13], the relevance of specific tests appears to vary [5,6,7,8,9,10,11,12] and evidence is not clear on which assessments are most insightful. It may also be impractical for non-researchers to acquire the more expensive, research-grade equipment needed to run traditional physiological assessments (e.g., metabolic cart, cycle ergometer, force plates). Instead, it may be easier to use standardized CrossFit^®^ workouts to monitor improvements. The annual CrossFit Games^TM^ competition sets out to find the fittest men and women through a series of stages, beginning with the CFO, and each stage features a unique battery of CrossFit^®^-style workouts [1,2,3]. Like the training style, each workout is designed to differentially challenge some combination of each athlete’s strength, endurance, and sport-specific skill [2,4]. After their introduction, CFO workouts become benchmarks that may be incorporated into training. Unlike everyday workouts, the standard requirements of each benchmark workout allow trainees to relate changes in their score to improvements in either the physiological traits or skills associated with the specific workout. Additionally, because CFO competitors are ranked by their performance in each workout [3], trainees might use their improvements in benchmark workouts to gauge how they might place in future CFO competitions.

Although workout performances are ranked in the CFO [3], several inherent flaws in the ranking process could lead to misinterpretation of where one truly ranks and in the degree of improvement needed to advance in rank. Within a specific CFO competition, athletes who complete a workout ‘as prescribed’ (i.e., Rx) are ranked, albeit higher, on the same scale as athletes who completed a scaled version [3]. That is, completing only a single repetition of the Rx workout will earn a higher rank than a record-setting performance in a scaled version of the same workout. Because this can improve their rank by several thousand places, athletes may attempt the Rx version knowing that they do not possess the skill or capacity to complete the entire workout or some of its components. Regardless, the inclusion of these well-below-average performances skews the calculation of a score’s associated percentile rank. Percentile ranks may also be skewed by the inclusion of “specialist” performances by athletes who only complete or submit scores for workouts that suit their strengths. While their performance in the specific workout that suited their skills may not be objectionable in itself, their extremely poor or non-existent performances in all other workouts make it difficult to affirm that they are part of the main competition’s population (i.e., athletes who capably completed all workouts within a single competition). Rather, because they could (or did) not complete all workouts, these athletes should be more closely associated with the scaled division populations. Likewise, athletes who intentionally (or unintentionally) fail to meet movement standards, miscount repetitions, or outright cheat, and still manage to successfully validate their score, cannot be viewed as being part of the main competition population. The presence of these scores adds too much variability to produce precise ranking thresholds from the entire pool of scores. Consequently, this study used very specific and standardized criteria to limit their inclusion before calculating any normative scores.

Rank-boosting performances skew the final population of scores [20] and lead to reduced thresholds distinguishing performance among higher percentile ranks. Previously, normative values were established from the self-reported scores for the five benchmark workouts that CFO competitors may upload to their user profiles [17]. In that study, exaggerated scores were addressed by uniform removal of all cases exceeding four SDs from the mean. This was problematic because SD is calculated from all scores [20] and uniformly removing scores based on its position on the normal curve will necessarily lead to illegitimate attempts causing a portion of valid attempts to be removed from both tails. Therefore, the present study used a different approach and only removed cases when the reported score did not exceed the minimum expectation for a legitimate attempt. This process still produced limitations because the minimum expectations were subjective creations and varied in degree of stringency depending on each workout’s programming. For instance, completing “one round” in AMRAP-style workouts resulted in the minimum expectation being as few as two repetitions (e.g., CFO 11.3 and CFO 12.1) to as many as 157 repetitions (i.e., CFO 15.3). When the threshold required athletes to complete the first exercise or exercise couplet, no greater ambiguity existed than when deciding what this criterion meant for CFO 20.5. In that workout, athletes could partition the workload (120 wall ball shots, 80 calories of rowing, and 40 ring muscle-ups) any way. Fortunately, pilot work suggested that within the top 10,000 competitors, legitimate attempts accumulated at least 80 repetitions between the rowing and wall ball shots but not necessarily both, and 40 muscle-ups would be the performance distinguishing factor [24]. These criteria still do not prevent legitimate attempts from being removed, and greater reliance is placed on the authors’ familiarity with the sport. Nevertheless, these criteria were consistently applied across all workouts and eliminated the arbitrary removal of elite performances, and it seems reasonable to assume that the resultant normative scores would still accurately place any valid, low-ranking (i.e., <1%) performances that were removed by this process.

Cases involving “specialists”, systematic reporting errors, and outright cheating also skew performances and lead to inflated thresholds distinguishing higher percentile ranks [20]. Like the previous normative study [17], these were dealt with by random athlete selection of the remaining cases [20]. This process does not guarantee the elimination of these cases but helps to reduce any systematic appearances to produce normative scores that are not artificially pulled in either direction. Although the success of these criteria can only be verified by a costly, international-scale, in-person study to repeat 11 years of CFO workouts, this does not seem to be necessary. The study criteria were designed to produce percentile scores that were relevant to and in line with the definition of the overall CrossFit^®^ ideology [4].

A secondary aim of this study was to compare performances by men and women across each CFO workout. The sport emphasizes gender equity in the number of male and female competitors invited to compete at the CrossFit Games^TM^, the monetary compensation [25], and the design of each workout [2,3]. Regarding the latter, CFO workouts are often scaled between sexes, presumably to elicit a similar challenge and account for known physiological differences. Theoretically, appropriate scaling should yield no differences between men and women in repetitions completed or TTC. CFO programming accomplished this by adjusting prescribed weight training exercise loads for women to be approximately 66.9 ± 4.4% of the weight assigned to men, or uniformly reducing women’s box height (for jumps, jump-overs, or step-ups) by ~17%, medicine ball weight by 30%, and wall ball shot target height by 10% from their respective prescriptions in men [2]. Such adjustments were present for at least one exercise in 55 (out of 60) CFO workouts. Still, sex differences were observed in 51 (of the 55 scaled workouts) and in all unscaled workouts (i.e., sex differences were noted in a total of 56 workouts).

Men are generally stronger than women [26]. Indeed, the *largest* performance differences were noted in the three workouts that required athletes to find their 1-RM (CFO 15.1a, CFO 18.2a, and CFO 21.4). CFO workouts presumably attempt to account for expected strength differences by adjusting weight training exercise loads (50 out of 60 workouts) and box height and wall ball shot criteria (18 out of 60 workouts). Even when the workout contained no purposefully scaled component, it may be argued that the design naturally accounted for strength differences. Body mass, which typically differs between men and women [26,27], altered the intensity of the only “unscaled” workouts that did not program 1-RMs (i.e., CFO 12.1 and CFO 21.1). Nevertheless, persistent differences in performance suggest that scaling was not sufficient to equate the challenge for most athletes. Without counting 1-RM workouts, ties were only noted in 7% (*n* = 4) of CFO workouts. Otherwise, men or women outperformed the other sex ~63% (*n* = 36) or ~30% (*n* = 17) of the time, respectively. Interestingly, average relative loads assigned to women varied from the average prescribed across all CFO workouts whenever either sex performed better. When men scored better than women, the loads assigned to women were slightly higher (68.3 ± 2.7% of loads assigned to men), and then slightly less (64.7 ± 4.0% of loads assigned to men) when women scored better than men. When men and women tied and the workouts involved a resistance training exercise (i.e., CFO 16.4, CFO 18.4, and CFO 20.3), relative loads prescribed to women (67.4 ± 2.1% of loads assigned to men) was closer to the average. However, the workouts only needed to adjust loads for one exercise, the deadlift. Thus, it may be hypothesized that the absolute loads assigned to men and women played a role in the observed performance differences and that ideal load scaling may vary based on the specific exercise.

Another programming aspect to consider is the lack of scaling for either the number of repetitions assigned to gymnastic–calisthenic exercises or the duration of traditional aerobic modalities. Besides the 1-RM workouts, men outperformed women by *large* differences in CFO 19.1, CFO 21.1, and CFO 21.3. While CFO 19.1 scaled wall ball shots and CFO 21.3 scaled front squat and thruster loads, all three workouts were 15 min long and involved unscaled, high-volume prescription for exercises that required upper-body muscular endurance (e.g., rowing, wall walks, muscle-ups, etc.). Likewise, in 7 of the 11 workouts where men scored better than women by a *medium* difference, the workout duration was between 10 and 20 min and included at least one high-volume, upper-body gymnastic exercise. Men are also known to possess greater aerobic and anaerobic capacity and more upper-body strength endurance than women [27,28,29], and not scaling these components may have contributed to them performing better. That said, there were two instances (CFO 15.1 and CFO 15.4) where unscaled, upper-body gymnastic exercises were programmed, and women outperformed men. However, both workouts also programmed 1–2 scaled, resistance training exercises that could have helped to offset any disadvantage they may have had from the toes-to-bar or handstand push-up exercises.

Men will typically outperform women whenever absolute values for traditional measures of strength and endurance are used, but not when these measures are standardized (e.g., percentage of 1-RM, per kilogram of body mass) [29,30,31]. Though it is beyond the scope of this study to speculate on the feasibility of relative programming or scaling gymnastic and aerobic components, these findings suggest CFO programming is regularly providing a different challenge to men and women. A counter argument is that it may be unnecessary, excessively tedious, and nearly impossible to equate CFO workout difficulty between sexes on an annual basis. Men and women do not directly compete, and a better performance from either sex will not impact their rankings [3]. Complicating prescription by assigning relative loads (e.g., percentages of established 1-RM) might create additional opportunities for cheating, and this would still not address traditionally unscaled components. It may also only be a matter of time before existing scaling methods naturally become regularly sufficient. Further analyses of data previously presented by Mangine [16] showed that women have experienced an ~8.3% improvement across all repeated CFO workouts compared with ~2.8% in similarly ranked men. Additionally, representation by women in the CFO has grown from 34.3% to 44.1% of all competitors in 2011 and 2022, respectively [19], and from 30.2% to 36.2% of all competitors meeting this study’s criteria. The combination of improved fitness and greater participation may naturally eliminate any regularity seen between sexes in CFO performance. Meanwhile, the purpose of the CFO is to identify the athletes who will be able to be competitive at later rounds (i.e., currently the top 10%) [3]. Manipulating prescription to equate the challenge when differences were predominantly (39 of 60 CFO workouts) *small*, *trivial*, or non-existent might be irrelevant to that purpose.

## 5. Conclusions

The present study created normative values for men and women in all CFO workouts. These data provide a current representation of the standards that distinguish performance in an ever-growing list of benchmark workouts. Periodic updates to account for changes in the population and new CFO workouts will undoubtedly be needed in the future. However, it is foreseeable that the list, currently at 60, will easily surpass 100 in the next decade and beyond, especially if traditional benchmark and “Hero” workouts are also considered. Such efforts may ultimately prove to be redundant and unnecessary. Recently, it was suggested that relationships might exist amongst workouts and/or workout components (e.g., the pull-up component of “Fran”) [21]. If true, workout components or entire workouts might be classified, and normative scores may only be necessary for symbolic representations of workout types or classifications. Currently, however, fair associations are impossible without the development of a simple and universal method for quantifying and equating workloads in any CrossFit^®^-style workout.

For the time being, the normative scores calculated in this study may be useful to CrossFit^®^ trainees and athletes for identifying strengths and weaknesses, assessing progress, and establishing realistic training goals. As research on this training strategy continues to grow, these values may help researchers to better identify individuals who are most representative of a targeted population. Existing studies have typically relied on the presence (or lack of) training experience (i.e., years of participation) to define a participant’s familiarity with CrossFit^®^ or high-intensity functional training. However, proficiency with the massive array of exercises that could potentially appear during a workout, as well as capability in regularly selecting appropriate pacing strategies, cannot be inferred simply from years of experience. In contrast, each year’s battery of CFO workouts was meant to, albeit variably, challenge aptitude across a broad range of sport-related traits and skills [2,3]. Not only are the selected exercises, movement standards, and prescriptions commonly incorporated into training, but standardized equipment makes it easier for most training facilities and laboratories to be adequately equipped for these workouts. Moreover, because CFO workouts are all designed to produce a score that distinguishes performance, these normative values can readily quantify individual skill in a single CFO workout or battery of workouts. Athletes, coaches, and researchers need only select the workouts that most closely resemble the needs of their training or study.

## Figures and Tables

**Table 1:** Population and sample characteristics.

	Women					Men				
Year	N_Total_	N_Study_	*n*	Age (y)	Rank (Range)	N_Total_	N_Study_	*n*	Age (y)	Rank (Range)
2011	4506	3046	2084	30.3 ± 6.4	2039 ± 1242 (1–4491)	8621	7046	4764	29.6 ± 6.3	4089 ± 2468 (1–8619)
2012	14,217	8621	9715	30.8 ± 5.8	4574 ± 2869 (3–12,089)	25,027	18,873	25,146	30.6 ± 5.8	9776 ± 5861 (1–21,861)
2013	32,643	14,144	5864	31.3 ± 6.9	8008 ± 5389 (1–25,127)	52,169	36,808	12,852	31.6 ± 7.1	19,177 ± 11,500 (1–45,181)
2014	52,076	36,863	18,174	31.1 ± 7	14,668 ± 9520 (1–42,021)	80,284	63,828	43,371	31.8 ± 7.2	32,570 ± 19,137 (1–70,402)
2015	108,764	7787	5313	29.7 ± 6.1	5000 ± 3866 (3–22,769)	153,272	45,615	31,006	30.7 ± 6.6	24,568 ± 15,552 (1–66,148)
2016	130,154	16,372	11,135	30.4 ± 6.4	9875 ± 7042 (1–35,593)	178,510	53,920	36,662	31.3 ± 6.7	28,396 ± 17,509 (1–76,110)
2017	159,563	36,721	25,096	31.8 ± 7.1	20,299 ± 13,286 (1–63,069)	214,519	84,669	57,311	32.6 ± 7.2	49,063 ± 32,387 (2–137,473)
2018	171,976	31,007	21,130	31.8 ± 7	17,926 ± 12,513 (1–63,422)	227,562	78,268	52,994	32.4 ± 7	44,822 ± 29,926 (2–138,037)
2019	146,363	39,895	39,895	32.8 ± 7.4	22,606 ± 15,224 (1–72,134)	195,562	87,197	87,197	33.9 ± 7.4	50,957 ± 33,750 (1–140,693)
2020	94,157	20,965	14,219	32.9 ± 7.4	12,358 ± 8831 (2–46,161)	133,874	51,394	34,932	33.7 ± 7.3	29,294 ± 19,543 (2–90,686)
2021	108,641	42,799	28,961	33.5 ± 7.8	22,449 ± 13,553 (4–53,595)	137,464	73,750	29,056	32.8 ± 7	21,715 ± 12,600 (1–43,847)
2022	122,177	51,011	34,675	33.4 ± 7.7	27,175 ± 16,814 (2–67,891)	154,815	89,792	61,055	34.5 ± 7.7	48,484 ± 30,324 (3–117,302)

**Table 2:** Programming and normative scores for 2011–2012 CFO workouts.

Programming				Percentile Rank												
Workout	Duration	Prescription	Sex	99	95	90	80	70	60	50	40	30	20	10	5	1
11.1 repetitions	10 min AMRAP	30 × Double-unders	W	355	315	304	270	261	244	224	214	196	175	148	131	94
		15 × Power snatches (75 lbs/55 lbs)	M	389	352	333	308	293	269	259	240	220	195	165	135	95
11.2 repetitions	15 min AMRAP	9 × Deadlifts (155 lbs/100 lbs)	W	507	437	410	376	354	338	324	304	288	269	246	225	188
		12 × Push-ups	M	511	453	430	398	373	353	336	321	303	284	260	237	197
		15 × Box jumps (24”/20”)														
11.3 repetitions	5 min AMRAP	1 × Squat clean (165 lbs/110 lbs)	W	71	59	52	44	38	33	29	24	19	12	5	1	1
		1 × Jerk (165 lbs/110 lbs)	M	73	63	58	50	45	40	36	31	26	20	12	5	1
11.4 repetitions	10 min AMRAP	60 × Bar-facing burpees	W	109	95	91	90	90	84	78	73	69	65	61	60	60
		30 × Overhead squats (120 lbs/90 lbs)	M	143	127	118	102	96	93	90	89	81	74	66	62	60
		10 × Ring muscle-ups														
11.5 repetitions	20 min AMRAP	5 × Power cleans (145 lbs/100 lbs)	W	360	318	304	278	260	246	233	216	205	185	157	132	90
		10 × Toes-to-bar	M	387	341	322	303	281	270	252	242	225	214	189	173	128
		15 × Wall ball shots (20 lbs/14 lbs to 10′/9′ target)														
11.6 repetitions	7 min AMRAP	3 × Thrusters (100 lbs/65 lbs)	W	126	110	103	94	86	81	76	71	61	55	41	29	12
		3 × Chest-to-bar pull-ups	M	137	125	117	107	101	96	90	85	79	72	64	54	32
		* Add 3 repetitions after each set														
12.1 repetitions	7 min AMRAP	Burpees	W	124	115	110	105	101	97	93	90	86	82	76	72	64
			M	134	124	119	113	108	104	101	96	92	87	80	75	66
12.2 repetitions	10 min AMRAP	30 × Snatches (75 lbs/45 lbs)	W	92	87	80	71	65	61	60	60	60	59	45	34	30
		30 × Snatches (135 lbs/75 lbs)	M	85	76	72	66	62	60	60	57	50	42	32	30	30
		30 × Snatches (165 lbs/100 lbs)														
		Max repetitions × Snatches (210 lbs/120 lbs)														
12.3 repetitions	18 min AMRAP	15 × Box jumps (24”/20”)	W	422	370	341	309	285	270	251	238	223	204	178	160	105
		12 × Push press (115 lbs/75 lbs)	M	421	375	349	315	294	275	260	243	231	211	193	168	129
		9 × Toes-to-bar														
12.4 repetitions	12 min AMRAP	150 × Wall ball shots (20 lbs/14 lbs to 10′/9′ target)	W	255	247	243	240	240	240	240	225	202	182	164	155	150
		90 × Double-unders	M	265	257	253	248	245	242	240	240	213	187	166	156	150
		30 × Muscle-ups														
12.5 repetitions	7 min AMRAP	3 × Thrusters (100 lbs/65 lbs)	W	126	111	104	91	85	80	75	68	58	52	34	28	13
		3 × Chest-to-bar pull-ups	M	137	123	115	104	99	93	87	82	76	70	61	54	31
		* Add 3 repetitions after each set														

* = Special instructions applied to specific workout’s prescription.

**Table 3:** Programming and normative scores for 2013–2014 CFO workouts.

Programming			Percentile Rank													
Workout	Duration	Prescription	Sex	99	95	90	80	70	60	50	40	30	20	10	5	1
13.1 repetitions	17 min AMRAP	*Alternate the following exercises:*	W	191	176	168	159	153	150	150	150	146	131	118	108	100
		40 → 30 → 20 → 10 × Burpees	M	174	163	158	151	150	141	128	122	115	108	101	100	100
		30 × Snatches at (75 lbs/45 lbs) → (135 lbs/75 lbs) → (165 lbs/100 lbs)														
		Then, max repetitions × Snatches (210 lbs/120 lbs)														
13.2 repetitions	10 min AMRAP	5 × Shoulder-to-overheads (115 lbs/75 lbs)	W	350	325	310	295	280	270	260	249	240	228	210	197	170
		10 × Deadlifts (115 lbs/75 lbs)	M	330	303	288	270	256	243	235	225.8	213	204	186	177	153
		15 × Box jumps (24”/20”)														
13.3 repetitions	12 min AMRAP	150 × Wall ball shots (20 lbs/14 lbs to 10′/9′ target)	W	257	249	245	240	240	240	240	240	215	193	170	158	150
		90 × Double-unders	M	266	258	254	248	244	241	240	235	206	183	164	155	150
		30 × Muscle-ups														
13.4 repetitions	7 min AMRAP	3 × Clean and jerk (135 lbs/95 lbs)	W	103	94	88	76	71	68	64	61	56	47	42	37	21
		3 × Toes-to-bar	M	108	100	95	87	79	73	70	67	63	60	48	43	35
		* Add 3 repetitions after each set														
13.5 repetitions	≥4 min AMRAP	15 × Thrusters (100 lbs/65 lbs)	W	144	84	78	70	61	57	54	51	49	46	42	38	30
		15 × Chest-to-bar pull-ups	M	152	130	87	78	72	68	64	60	55	51	46	42	35
		* Add 4 min each time 3 sets are completed within time limit														
14.1 repetitions	10 min AMRAP	30 × Double-unders	W	371	341	311	297	267	258	235	220	210	179	150	129	90
		15 × Power snatches (75 lbs/55 lbs)	M	381	348	316	303	273	262	249	224	211	179	142	121	90
14.2 repetitions	3 min rounds (indefinite)	*Complete 2 sets of:*	W	203	143	134	114	82	77	68	59	36	33	29	24	20
		10 × Overhead squats (95 lbs/65 lbs)	M	254	194	175	133	122	109	82	76	69	59	34	27	20
		10 × Chest-to-bar pull-ups														
		* Add 3 min and 2 repetitions after each set														
14.3 repetitions	8 min AMRAP	*Alternate the following exercises:*	W	158	147	141	135	130	119	110	106	102	97	91	90	62
		Deadlifts: 10 × (135 lbs/95 lbs) → 15 × (185 lbs/135 lbs) → 20 × (225 lbs/155 lbs) → 25 × (275 lbs/185 lbs) → 30 × (315 lbs/205 lbs) → 35 × (365 lbs/225 lbs)	M	152	143	138	132	130	117	110	106	102	98	93	90	69
		15 × Box jumps (24”/20”)														
14.4 repetitions	14 min AMRAP	60-calorie Rowing	W	191	184	181	180	180	176	170	164	159	153	141	124	93
		50 × Toes-to-bar	M	213	194	190	185	182	180	180	177	171	164	156	146	102
		40 × Wall ball shots (20 lbs/14 lbs to 10′/9′ target)														
		30 × Cleans (135 lbs/95 lbs)														
		20 × Ring muscle-ups														
14.5 TTC	No time limit	*21 → 18 → 15 → 12 → 9 → 6 → 3 repetitions:*	W	10:39	11:01	12:51	14:01	15:01	16:01	16:01	17:01	19:01	20:01	22:01	25:21	30:01
		Thrusters (95 lbs/65 lbs)	M	10:40	12:01	13:01	14:33	15:01	16:01	17:01	19:01	20:01	22:21	25:01	27:01	34:01
		Burpees														

* = Special instructions applied to specific workout’s prescription.

**Table 4:** Programming and normative scores for 2015–2016 CFO workouts.

Programming			Percentile Rank													
Workout	Duration	Prescription	Sex	99	95	90	80	70	60	50	40	30	20	10	5	1
15.1 repetitions	9 min AMRAP	15 × Toes-to-bar	W	218	205	191	182	175	162	158	154	149	136	127	120	98
		10 × Deadlifts (115 lbs/75 lbs)	M	211	190	182	166	158	152	147	136	129	124	117	103	90
		5 × Snatches (115 lbs/75 lbs)														
	6 min time limit	*Immediately into:*	W	220 (99.8)	202 (91.6)	193 (87.5)	181 (82.1)	175 (79.4)	165 (74.8)	160 (72.6)	155 (70.3)	145 (65.8)	140 (63.5)	134 (60.8)	125 (56.7)	115 (52.2)
15.1 a lbs. (kg)		1-RM Clean and jerk	M	316 (143.5)	290 (131.5)	275 (124.7)	255 (115.7)	245 (111.1)	235 (106.6)	225 (102.1)	215 (97.5)	205 (93)	198 (89.8)	185 (83.9)	176 (79.8)	165 (74.8)
15.2 repetitions	3 min rounds (indefinite)	*Complete 2 sets:*	W	278	254	202	192	172	138	133	128	117	86	80	75	63
		10 × Overhead squats (95 lbs/65 lbs)	M	277	244	199	179	140	134	127	118	109	83	74	67	56
		10 × Chest-to-bar pull-ups														
		* Add 3 min and 2 repetitions after each set														
15.3 repetitions	14 min AMRAP	7 × Ring muscle-ups	W	478	447	371	332	318	315	279	211	171	161	158	157	157
		50 × Wall ball shots (20 lbs/14 lbs to 10′/9′ target)	M	504	474	436	362	340	320	316	301	236	190	160	158	157
		100 × Double-unders														
15.4 repetitions	8 min AMRAP	3 × Handstand push-ups	W	143	124	111	98	89	80	73	66	57	50	37	30	16
		3 × Cleans (185 lbs/125 lbs)	M	128	106	95	80	71	64	56	51	45	36	28	20	10
		* Add 3 repetitions after each set														
15.5 TTC	No time limit	*27 → 21 → 15 → 9 repetitions:*	W	7:18	7:59	8:28	9:40	9:38	10:56	10:36	11:50	11:40	12:28	13:39	14:46	17:27
		Rowing (calories)	M	6:29	7:25	7:59	8:51	9:31	10:06	10:44	11:24	12:10	13:06	14:33	15:54	19:40
		Thrusters (95 lbs/65 lbs)														
16.1 repetitions	20 min AMRAP	25 ft Overhead walking lunge (95 lbs/65 lbs)	W	289	260	239	219	206	194	183	174	166	156	143	130	108
		8 × Burpees	M	291	260	240	219	206	193	182	171	163	153	136	124	104
		25 ft Overhead walking lunge (95 lbs/65 lbs)														
		8 × Chest-to-bar pull-ups														
16.2 repetitions	4 min rounds (20 min time limit)	25 × Toes-to-bar	W	425	343	339	259	255	253	176	173	171	168	165	154	119
		50 × Double-unders	M	346	339	260	255	234	175	172	170	168	166	165	144	114
		Squat cleans: 15 × (135 lbs/85 lbs) → 13 × (185 lbs/115 lbs) → 11 × (225 lbs/145 lbs) → 9 × (275 lbs/175 lbs) 7 × (315 lbs/205 lbs)														
		* Add 4 min for each completed set														
16.3 repetitions	7 min AMRAP	10 × Power snatches (75 lbs/55 lbs)	W	117	103	95	86	76	70	63	53	49	37	25	24	23
		3 × Bar muscle-ups	M	123	111	103	96	89	86	78	75	69	62	49	37	23
16.4 repetitions	13 min AMRAP	55 × Deadlifts (225 lbs/155 lbs)	W	257	229	211	199	191	185	181	177	172	167	159	146	114
		55 × Wall ball shots (20 lbs/14 lbs to 10′/9′)	M	256	225	209	197	190	185	181	177	173	169	165	149	111
		55-calorie Rowing														
		55 × Handstand push-ups														
16.5 TTC	No time limit	*21 → 18 → 15 → 12 → 9 → 6 → 3 repetitions:*	W	9:16	10:22	11:06	12:30	12:49	13:30	14:09	14:47	15:34	16:34	18:30	19:17	22:20
		Thrusters (95 lbs/65 lbs)	M	9:27	10:48	11:42	12:51	13:45	14:35	15:22	16:14	17:10	18:23	20:20	21:47	25:21
		Burpees														

* = Special instructions applied to specific workout’s prescription.

**Table 5:** Programming and normative scores for 2017–2018 CFO workouts.

Programming			Percentile Rank													
Workout	Duration	Prescription	Sex	99	95	90	80	70	60	50	40	30	20	10	5	1
17.1 TTC → repetitions	20 min time limit	*Alternate the following:*	W	12:15	13:54	14:59	16:23	17:25	18:22	19:11	19:53	215	203	183	170	149
		Dumbbell snatches (50 lbs/35 lbs): × 10 → 20 → 30 → 40 → 50 repetitions	M	12:50	13:51	14:58	16:30	17:39	18:38	19:29	220	211	195	177	164	145
		15 × Burpee box jump-overs (24”/20”)														
17.2 repetitions	12 min AMRAP	*Complete 2 sets:*	W	182	141	122	113	91	85	80	78	78	78	78	78	73
		50 ft Walking dumbbell lunges (50 lbs/35 lbs)	M	194	163	146	125	118	114	106	90	85	80	78	78	76
		16 × Toes-to-bar														
		8 × Dumbbell power cleans (50 lbs/35 lbs)														
		*Then, complete 2 sets:*														
		50 ft Walking dumbbell lunges (50 lbs/35 lbs)														
		16 × Bar muscle-ups														
		8 × Dumbbell power cleans (50 lbs/35 lbs)														
17.3 TTC → repetitions	8 min AMRAP (24 min time limit)	*Prior to 8 min, complete 3 sets:*	W	154	105	92	80	68	59	55	47	44	43	43	41	36
		6 × Chest-to-bar pull-ups	M	167	119	105	92	80	71	65	57	52	45	43	43	38
		6 × Squat snatches (95 lbs/65 lbs)														
		*Then, complete 3 sets:*														
		7 × Chest-to-bar pull-ups														
		5 × Squat snatches (135 lbs/95 lbs)														
		* Add 4 min after completing 3 sets:														
		8 → 9 → 10 → 11 × Chest-to-bar pull-ups														
		Squat snatches: 4 × (185 lbs/135 lbs) → 3 × (225 lbs/155 lbs) → 2 × (245 lbs/175 lbs) → 1 × (265 lbs/185 lbs)														
17.4 repetitions	13 min AMRAP	55 × Deadlifts (225 lbs/155 lbs)	W	256	218	203	190	183	177	173	169	165	165	149	134	98
		55 × Wall ball shots (20 lbs/14 lbs to 10′/9′ target)	M	260	226	208	195	187	181	177	173	168	165	149	131	95
		55-calorie Rowing														
		55 × Handstand push-ups														
17.5 TTC → repetitions	40 min time limit	Complete 10 sets of:	W	8:06	9:33	10:34	12:50	13:20	14:31	15:46	17:10	18:53	21:30	26:25	32:24	323
		9 × Thrusters (95 lbs/65 lbs)	M	8:20	9:40	10:48	12:31	13:58	15:25	16:56	18:38	20:46	24:40	30:33	38:47	265
		35 × Double-unders														
18.1 repetitions	20 min AMRAP	8 × Toes-to-bar	W	379	348	329	307	291	278	268	257	245	232	215	200	175
		10 × Dumbbell hang clean and jerks (50 lbs/35 lbs)	M	425	391	370	347	330	320	305	292	279	264	245	232	203
		14-calorie Rowing														
18.2 TTC → repetitions	12 min time limit	*Complete 1 → 2 → 3 → 4 → 5 → 6 → 7 → 8 → 9 → 10 repetitions:*	W	4:46	5:31	5:58	6:34	6:58	7:22	7:43	8:50	8:31	9:00	9:47	10:21	11:15
		Dumbbell squats (50 lbs/35 lbs)	M	4:35	5:22	5:49	6:25	6:53	7:18	7:43	8:08	8:36	9:10	9:56	10:32	11:25
		Bar-facing burpees														
18.2 a lbs. (kg)		*Then:*	W	225 (102.1)	205 (93)	192 (87.1)	178 (80.7)	170 (77.1)	161 (73)	155 (70.3)	147 (66.7)	142 (64.4)	135 (61.2)	125 (56.7)	115 (52.2)	100 (45.4)
		1-RM Clean	M	335 (152)	305 (138.3)	287 (130.2)	267 (121.1)	255 (115.7)	243 (110.2)	232 (105.2)	225 (102.1)	212 (96.2)	200 (90.7)	185 (83.9)	175 (79.4)	154 (69.9)
18.3 TTC → repetitions	14 min time limit	*Complete 2 sets:*	W	689	578	486	432	230	224	220	220	220	220	220	220	162
		100 × Double-unders	M	722	675	584	536	462	453	380	302	227	222	220	220	152
		20 × Overhead squats (115 lbs/80 lbs)														
		100 × Double-unders														
		12 × Ring muscle-ups														
		100 × Double-unders														
		20 × Dumbbell snatches (50 lbs/35 lbs)														
		100 × Double-unders														
		12 × Bar muscle-ups														
18.4 TTC → repetitions	9 min time limit	*Complete 21-15-9 repetitions:*	W	164	136	119	111	103	96	89	83	70	65	60	58	48
		Deadlifts (225 lbs/155 lbs)	M	155	131	118	109	101	96	90	85	72	66	61	59	50
		Handstand push-ups														
		*Then, complete 21-15-9 repetitions:*														
		Deadlifts (315 lbs/205 lbs)														
		Handstand walk (50′)														
18.5 repetitions	7 min AMRAP	3 × Thrusters (100 lbs/65 lbs)	W	160	137	123	111	104	97	90	85	81	77	67	56	33
		3 × Chest-to-bar pull-ups	M	157	137	126	114	106	101	96	90	86	81	74	69	60
		*Add 3 repetitions after each set														

* = Special instructions applied to specific workout’s prescription.

**Table 6:** Programming and normative scores for 2019–2020 CFO workouts.

Programming			Percentile Rank													
Workout	Duration	Prescription	Sex	99	95	90	80	70	60	50	40	30	20	10	5	1
19.1 repetitions	15 min AMRAP	19 × Wall ball shots (20 lbs/14 lbs to 10′/9′ target)	W	309	287	273	258	248	239	229	222	213	204	190	177	154
		19-calorie Rowing	M	354	327	313	295	284	270	261	249	240	228	210	198	171
19.2 TTC → repetitions	4 min rounds (20 min time limit)	25 × Toes-to-bar	W	424	339	259	253	175	171	167	165	137	111	101	93	82
		50 × Double-unders	M	345	263	258	253	174	171	168	166	156	115	101	90	81
		Squat cleans: 15 × (135 lbs/85 lbs) → 13 × (185 lbs/115 lbs) → 11 × (225 lbs/145 lbs) → 9 × (275 lbs/175 lbs) 7 × (315 lbs/205 lbs)														
		* Add 4 min for each set														
19.3 TTC → repetitions	10 min time limit	200 ft Dumbbell overhead lunge (50 lbs/35 lbs)	W	159	134	120	107	98	93	90	90	90	90	90	87	59
		50 × Dumbbell box step-ups (50 lbs/35 lbs onto 24”/20” box)	M	161	140	129	118	111	105	100	96	92	90	86	72	52
		50 × Strict handstand push-ups														
		200 ft Handstand walking														
19.4 TTC → repetitions	12 min time limit	*Complete 3 sets:*	W	11:26	115	110	93	88	72	67	66	66	66	66	66	66
		10 × Snatches (95 lbs/65 lbs)	M	10:24	11:44	121	113	110	97	93	90	76	70	66	66	66
		12 × Bar-facing burpees														
		*Then, rest 3 min before completing 3 sets:*														
		10 × Bar muscle-ups														
		12 × Bar-facing burpees														
19.5 TTC → repetitions	20 min time limit	*Complete 33 → 27 → 21 → 15 → 9 repetitions:*	W	11:28	15:09	17:14	19:46	190	179	163	152	143	126	107	98	83
		Thrusters (95 lbs/65 lbs)	M	11:07	14:12	16:14	18:47	203	187	177	165	153	142	127	113	89
		Chest-to-bar pull-ups														
20.1 TTC → repetitions	15 min time limit	*Complete 10 sets:*	W	10:16	11:50	12:44	13:49	14:33	178	168	162	154	147	136	128	113
		8 × Ground-to-overheads (95 lbs/65 lbs)	M	10:27	12:09	13:06	14:11	14:50	170	164	157	149	144	131	126	109
		10 × Bar-facing burpees														
20.2 repetitions	20 min AMRAP	4 × Dumbbell thrusters (50 lbs/35 lbs)	W	851	740	684	616	577	538	510	476	442	393	340	280	194
		6 × Toes-to-bar	M	855	751	691	636	586	549	515	481	446	408	341	281	198
		24 × Double-unders														
20.3 TTC → repetitions	9 min time limit	*Complete 21-15-9 repetitions:*	W	8:39	139	124	112	105	97	90	84	70	65	60	57	47
		Deadlifts (225 lbs/155 lbs)	M	163	134	122	111	103	97	92	87	80	67	61	58	50
		Handstand push-ups														
		*Then, complete 21-15-9 repetitions:*														
		Deadlifts (315 lbs/205 lbs)														
		Handstand walk (50′)														
20.4 TTC → repetitions	20 min time limit	*Alternate the following:*	W	17:55	235	235	201	200	200	169	163	160	160	160	130	120
		30 × Box jumps (24”/20”)	M	237	215	201	200	177	166	162	160	160	151	127	121	120
		Clean and jerks: 15 × (95 lbs/65 lbs) → 15 × (135 lbs/85 lbs) → 10 × (185 lbs/115 lbs.)														
		*Then, alternate the following:*														
		30 × Single-leg squats														
		Clean and jerks: 10 × (225 lbs/145 lbs) → 5 × (275 lbs/175 lbs) → 5 × (315 lbs/205 lbs.)														
20.5 TTC → repetitions	20 min time limit	*Partition in any way:*	W	15:16	19:50	230	217	209	178	168	162	154	147	136	128	113
		40 × Ring muscle-ups	M	12:27	14:09	16:06	18:11	237	170	164	157	149	144	131	126	109
		80-calorie Rowing														
		120 × Wall ball shots (20 lbs/14 lbs to 10′/9′ target)														

* = Special instructions applied to specific workout’s prescription.

**Table 7:** Programming and normative scores for 2021–2022 CFO workouts.

Programming			Percentile Rank													
Workout	Duration	Prescription	Sex	99	95	90	80	70	60	50	40	30	20	10	5	1
21.1 TTC → repetitions	15 min time limit	*Alternate the following:*	W	588	395	387	379	355	283	222	217	213	182	115	110	44
		Wall walks × 1 → 3 → 6 → 9 → 15 → 21 repetitions	M	14:16	505	415	389	384	381	378	374	332	277	221	217	210
		Double-unders × 10 → 30 → 60 → 90 → 150 → 210 repetitions														
21.2 TTC → repetitions	20 min time limit	*Alternate the following:*	W	11:14	12:51	13:52	15:15	16:26	17:32	18:35	19:36	217	201	177	160	131
		Dumbbell snatches (50 lbs/35 lbs): × 10 → 20 → 30 → 40 → 50 repetitions	M	10:47	11:57	12:44	13:47	14:35	15:18	15:59	16:41	17:26	18:18	19:21	19:59	205
		15 × Burpee box jump-overs (24”/20”)														
21.3 TTC → repetitions	15 min time limit	15 × Front squats (95 lbs/65 lbs)	W	11:14	12:51	158	146	139	135	135	135	120	95	77	75	75
		30 × Toes-to-bar	M	10:47	11:57	12:44	13:47	166	159	155	151	147	143	139	136	135
		15 × Thrusters (95 lbs/65 lbs)														
		*Rest 1 min, then:*														
		15 × Front squats (95 lbs/65 lbs)														
		30 × Chest-to-bar pull-ups														
		15 × Thrusters (95 lbs/65 lbs)														
		*Rest 1 min, then:*														
		15 × Front squats (95 lbs/65 lbs)														
		30 × Bar muscle-ups														
		15 × Thrusters (95 lbs/65 lbs)														
21.4 lbs (kg)	7 min time limit	*After 15 min time limit:*	W	197 (89.4)	176 (79.8)	165 (74.8)	154 (69.9)	145 (65.8)	135 (61.2)	130 (59)	125 (56.7)	117 (53.1)	110 (49.9)	101 (45.8)	95 (43.1)	85 (38.6)
		1-RM Complex of Deadlift → Clean → Hang clean → Jerk	M	292 (132.4)	267 (121.1)	255 (115.7)	238 (108)	227 (103)	220 (99.8)	211 (95.7)	205 (93)	198 (89.8)	187 (84.8)	177 (80.3)	167 (75.7)	154 (69.9)
22.1 repetitions	15 min AMRAP	3 × Wall walks	W	317	283	270	242	228	212	202	184	179	154	138	121	80
		12 × Dumbbell snatches (50 lbs/35 lbs)	M	316	289	272	248	240	219	211	196	182	167	150	125	92
		15 × Box jump-overs (24”/20”)														
22.2 TTC → repetitions	10 min time limit	*1 → 2 → 3 → 4 → 5 → 6 → 7 → 8 → 9 → 10 → 9 → 8 → 7 → 6 → 5 → 4 → 3 → 2 → 1 repetitions of:*	W	9:10	180	169	157	147	139	132	125	117	110	95	83	57
		Deadlifts (225 lbs/155 lbs)	M	9:36	183	171	158	149	141	133	126	117	111	96	89	62
		Bar-facing burpees														
22.3 TTC → repetitions	12 min time limit	21 × Pull-ups	W	6:10	9:34	11.2	169	161	156	156	156	154	132	89	84	84
		42 × Double-unders	M	6:36	8:23	9:39	11.55	208	183	165	160	156	156	142	113	86
		21 × Thrusters (95 lbs/65 lbs)														
		18 × Chest-to-bar pull-ups														
		36 × Double-unders														
		18 × Thrusters (115 lbs/75 lbs)														
		15 × Bar muscle-ups														
		30 × Double-unders														
		15 × Thrusters (135 lbs/85 lbs)														

## Data Availability

All data are publicly available at: https://games.crossfit.com/leaderboard/open/ (accessed on 1 December 2022).
